# Supplementary material for: Influence of bone microstructure on ultrasound loss through skull‐mimicking digital phantoms
Source: Med Phys. 2025 Oct 30;52(11):e70113. doi: 10.1002/mp.70113 (PMC12575882; doi:10.1002/mp.70113)
Supplement: Supplementary file 1 — Supporting Information [file MP-52-0-s001.pdf]

Supplemental Materials

Digital Phantoms

This section describes how digital phantoms with varying porosity and pore size were generated. Our goal was to have a set of phantoms with a range of porosity from 0% to 75% comprised of different pore diameters. We chose six pore diameters (0.2 mm, 0.3 mm, 0.4 mm, 0.6 mm, 0.8 mm, and 1.0 mm), under a wavelength (650 kHz, 2.3 mm) in water. Because the pores overlap randomly, no one-to-one correspondence exists between the number of pores placed and the porosity. To overcome this, eight target porosities (2.5%, 5%, 10%, 20%, 30%, 40%, 50%, and 75%) were chosen and achieved through an iterative process. For each pore diameter, the number of pores corresponding to a 0.5% increase in porosity, assuming no overlap, was determined by supplemental equation 1.

$$N_{0.5\%} = 0.05 * \frac{3\,TV}{4\pi r^3} \qquad \text{(S.1)}$$

Then, this number of pores was randomly placed repeatedly, and the porosity was calculated after each iteration. The process was complete when the porosity was equal to or greater than the target porosity. The randomization was completed by a uniformly random vector permutation of vectors expressing every possible location within the grid. Then, pore locations were chosen from the permuted vector so that no location could be repeated.

The actual porosity could exceed the target porosity by a maximum of 0.5%, as that is the maximum number of pores placed at a time. Due to this slight variation, we will refer to the target porosities as nominal porosities. Five phantoms with different random pore positions were created for statistical analysis at each nominal porosity and pore size.

CT Phantoms

We constructed CT-based phantoms using segmented temporal, frontal, and parietal bone from a CT scan. A clinical CT skull image of an 86-year-old male treated for essential tremor was retrospectively obtained (University of Utah, IRB 00121352). The CT was acquired on a Siemens SOMATOM Edge Plus scanner with 120 kVp, an H60 kernel, 1 mm slice resolution, and 0.5 mm in-plane resolution.

The temporal, frontal, and parietal bone sections were manually segmented (approximately 40 mm × 40 mm × 20 mm) and resampled to 0.05 mm isotropic resolution, matching the previously used phantom resolution. The CTs were rotated to align the outer table parallel to the slice dimension. A threshold segmentation (>100 HUs) was applied to distinguish bone from air and brain, with any holes filled using MATLAB's imfill function, resulting in a solid bone-shaped mask. The lateral extents of the volume were cropped to 25.6 mm × 25.6 mm, ensuring the bone mask filled the volume. This corresponds to a lateral grid size of 512x512, which is computationally efficient within k-Wave. Finally, 1 mm of the outer and inner tables were removed using MATLAB's imerode function with a spherical kernel to reduce the cortical regions.

Microstructure was added with porosities of 10%, 30%, and 50%, consisting of pore diameters of 0.2 mm, 0.4 mm, and 0.6 mm. The previously defined phantoms (12.6 mm × 12.6 mm × 5 mm) were replicated to form a larger phantom volume, which was then masked using the segmented bone structure. The final phantom model retained curved interfaces based on real bone while having a defined microstructure.

Figure S1 shows central longitudinal slices through 30% phantoms consisting of 0.2 mm or 0.6 mm pore diameters.

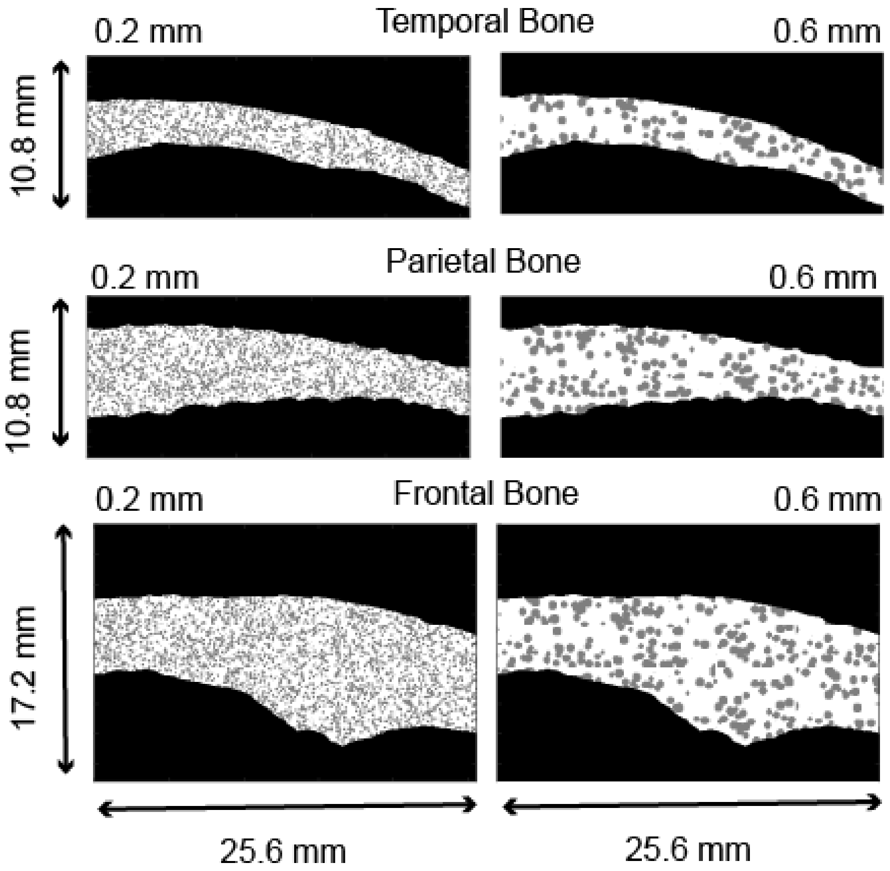

Supplemental Figure 1: CT-based phantoms with temporal, parietal, and frontal bone shapes. The idealized microstructure consists of spherical marrow pores in gray with 0.2 mm (left) or 0.6 mm (right) pore diameters randomly placed into a cortical background (white). The porosity is 30%. The black background corresponds to water.

Convergence Testing

Convergence testing was completed over the time duration and time step using the 9 phantoms shown in Figure 1, with representative pore diameters (0.2 mm, 0.6mm, 1.0 mm) and porosities (10%, 30%, 50%). The percentage difference of the mean steady-state pressure in the measurement plane with the test parameters compared to the reference parameters was used as a metric for convergence. We report the maximum percentage difference across the nine test phantoms for each test in red. Our paper reports these maximum differences, which are all well below 10%, indicating good convergence in all cases. The full results are reported in Supplemental Table 1.

First, we increased the simulation's duration by 2 times to 68 μs at 230 kHz and 54 μs at 650 kHz, resulting in a maximum difference of 9.045% and 3.711%, respectively. We inferred time duration convergence for absorbing phantoms because the additional absorption will cause the system to approach a steady state faster. Next, the CFL was reduced by a factor of 2 to 0.2 in the non-absorbing simulations and 0.01 and 0.03 in the absorbing simulation at 230 kHz and 650 kHz. The maximum percentage difference was 0.113% and 0.165% in the non-absorbing simulations at 230 kHz and 650 kHz. The maximum percentage difference was 0.031% and 0.003% in the absorbing simulations at 230 kHz and 650 kHz. We did not conduct convergence testing in the grid spacing, as our grid spacing results in 44 points per wavelength in the component with the minimum velocity at 650 kHz, which is well above the recommended minimum of 4 points per wavelength.

Supplemental Table 1: Convergence Test Results Through Nine Representative Phantoms

Phantom Porosity, Pore Diameter)	230 kHz Non-Absorbing CFL	650 kHz Non-Absorbing CFL	230 kHz Absorbing CFL	650 kHz Absorbing CFL	230 kHz Non-Absorbing Duration	650 kHz Non-Absorbing Duration
10%, 0.2 mm	0.055	0.006	0.017	0.002	2.467	0.386
30%, 0.2 mm	0.028	0.031	0.031	0.001	2.697	0.731
50%, 0.2 mm	0.027	0.164	0.031	0.003	0.733	3.711
10%, 0.6 mm	0.086	0.022	0.018	0.003	3.308	1.739
30%, 0.6 mm	0.024	0.005	0.017	0	3.665	1.865
50%, 0.6 mm	0.03	0.014	0.026	0	9.045	1.763
10%, 1.0 mm	0.113	0.024	0.017	0.001	0.065	2.941
30%, 1.0 mm	0.02	0.009	0.012	0.001	2.622	0.932
50%, 1.0 mm	0.026	0.022	0.023	0	8.113	0.623

Insertion Loss Vs. Density and Hounsfield Units

The primary result reported in Figure 4 shows the insertion loss through non-absorbing phantoms as a function of porosity. In literature, ultrasound loss (attenuation) is typically related to bone density or computed tomography (CT) Hounsfield Units (HU). Here, we report insertion loss as a function of density and HUs in supplemental Figures S2 and S3. This enables a more direct comparison to attenuation relationships found in the literature, as the linear transformations result in a horizontal axis flip, placing the low porosity phantoms at higher density and HU values.

In Figure S2, we assume density is linear with porosity, according to supplemental Equation S1 and as reported in Equation 4 in Aubry et al. 2003. We used the densities reported in Table 1 for marrow and cortical bone, 1029 kg/m^3 and 1908 kg/m^3, respectively. Other studies have used higher values for cortical bone density, for example, 2700 kg/m^3 in Marsac et al. 2017; 2100 kg/m^3 in Aubry et al. 2003; and 2200 kg/m^3 in Marquet et al. 2009.

$$\rho = \emptyset\rho_{min} + (1 - \emptyset)\rho_{max} \quad \text{(S1)}$$

In Figure S3, we assume that the CT HUs is also linear with porosity and density, as described by supplemental Equation S2 and reported in Equation 4 in Marsac et al. 2017. In that study, they used a HU minimum of -1024 HU and a maximum of 2400 HU. The exact minimum and maximum HUs depend on the CT parameters and the individual skull density. Leung et al. 2019 reports several literature attenuation relationships in the range from 0 to 2000 HU. Here, we assume the same range as Leung et al. 2019.

$$\text{HU} = (HU_{max} - HU_{min})\frac{\rho - \rho_{min}}{\rho_{max} - \rho_{min}} + HU_{min} \quad \text{(S2)}$$

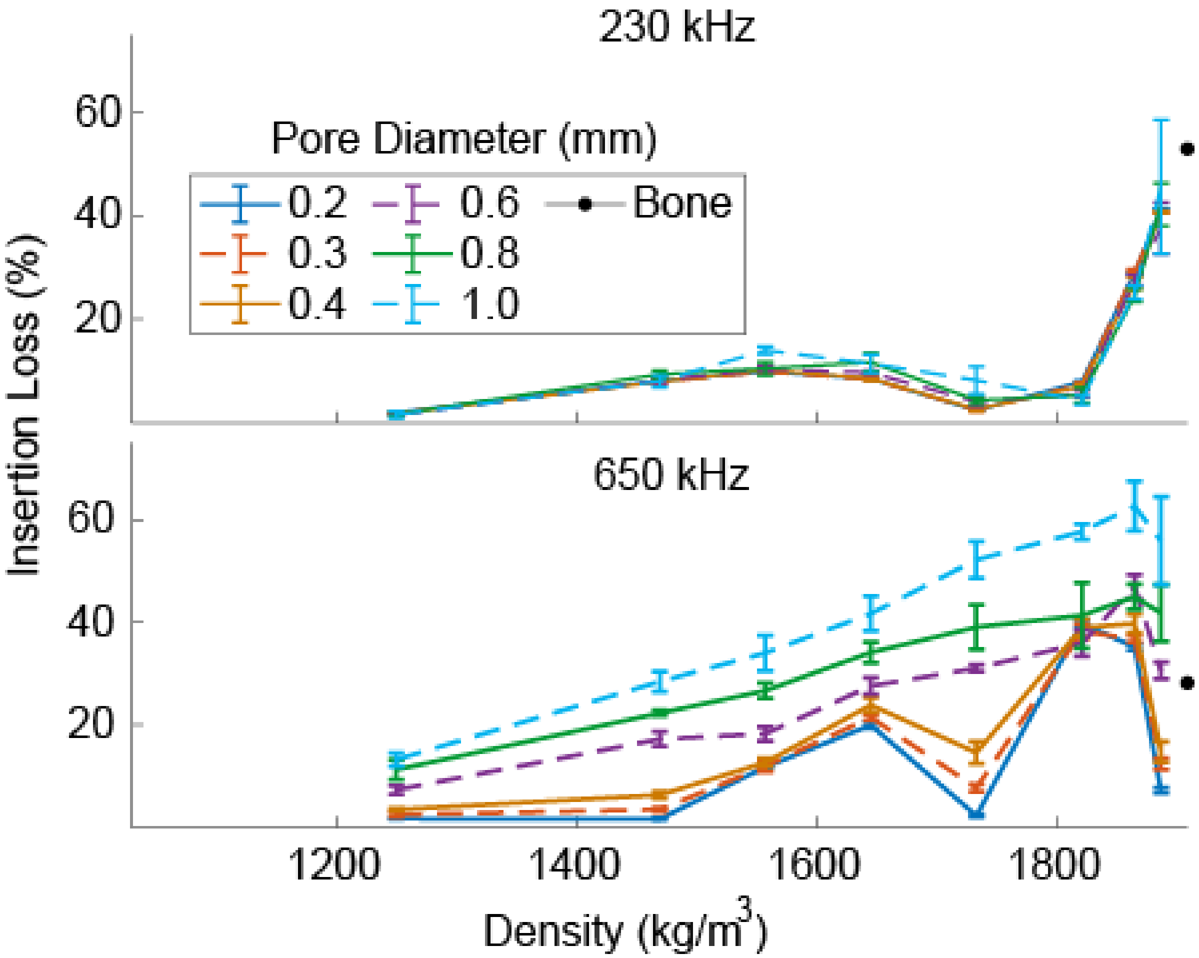

Supplemental Figure S2: Insertion loss through non-absorbing phantoms as a function of density assuming a linear relationship to porosity.

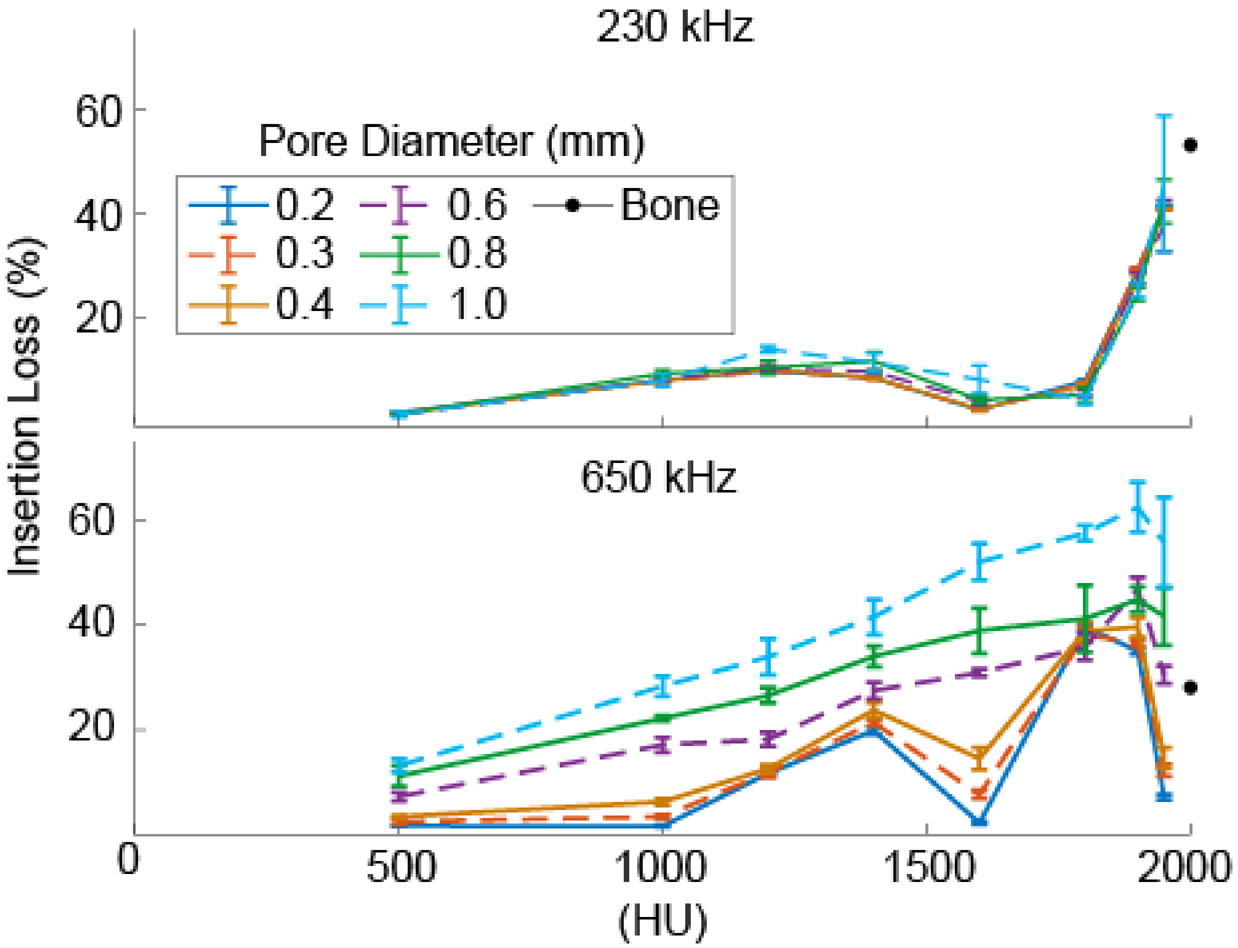

Supplemental Figure S3: Insertion loss through non-absorbing phantoms as a function of Hounsfield Units, assuming a linear relationship to density and porosity.

Supplemental References

Aubry, J.-F., Tanter, M., Pernot, M., Thomas, J.-L., & Fink, M. (2003). Experimental demonstration of noninvasive transskull adaptive focusing based on prior computed tomography scans. *The Journal of the Acoustical Society of America*, *113*(1), 84–93. https://doi.org/10.1121/1.1529663

Leung, S. A., Webb, T. D., Bitton, R. R., Ghanouni, P., & Butts Pauly, K. (2019). A rapid beam simulation framework for transcranial focused ultrasound. *Scientific Reports*, *9*(1), 1–11. https://doi.org/10.1038/s41598-019-43775-6

Marquet, F., Pernot, M., Aubry, J. F., Montaldo, G., Marsac, L., Tanter, M., & Fink, M. (2009). Non-invasive transcranial ultrasound therapy based on a 3D CT scan: Protocol validation and in vitro results. *Physics in Medicine and Biology*, *54*(9), 2597–2613. https://doi.org/10.1088/0031-9155/54/9/001

Marsac, L., Chauvet, D., La Greca, R., Boch, A. L., Chaumoitre, K., Tanter, M., & Aubry, J. F. (2017). Ex vivo optimisation of a heterogeneous speed of sound model of the human skull for non-invasive transcranial focused ultrasound at 1 MHz. *International Journal of Hyperthermia*, *33*(6). https://doi.org/10.1080/02656736.2017.1295322
